# Physiological mechanism of sodium salicylate and folcisteine on alleviating salt stress in wheat seedlings

**DOI:** 10.1038/s41598-023-49629-6

**Published:** 2023-12-18

**Authors:** Aohui Han, Cuiling Wang, Jingchong Li, Li Xu, Xiaoyan Guo, Weiguo Li, Feng Zhou, Runqiang Liu

**Affiliations:** 1 Henan Engineering Research Center of Green Pesticide Creation and Pesticide Residue Monitoring By Intelligent Sensor, Henan Institute of Science and Technology, Xinxiang, 453003 China; 2 Baiquan Institute of Advanced Agricultural Technology, Henan Institute of Science and Technology, Xinxiang, 453003 China; 3 School of Basic Medical Sciences, Xinxiang Medical University, Xinxiang, 453003 China

**Keywords:** Physiology, Plant sciences

## Abstract

Soil salinization substantially hampers the growth and development of wheat, potentially leading to plant death in severe cases, thus reducing grain yield and quality. This phenomenon poses a significant threat to food security in China. We investigated the effects of two exogenous plant growth regulators, sodium salicylate and folcisteine, on the wheat physiology and key characteristics under salt stress using hydroponics method. The results indicated that both regulators effectively mitigated the growth inhibition of wheat under salt stress. We assessed morphological and physiological indexes, including antioxidant enzyme activities (superoxide dismutase [SOD], catalase [CAT], peroxidase [POD]) and malondialdehyde (MDA) concentration in wheat after foliar application of sodium salicylate and folcisteine under salt stress. The findings revealed that sodium salicylate was more effective than folcisteine. However, folcisteine showed superior performance in reducing hydrogen peroxide (H_2_O_2_) content and superoxide anion (O^2−^) level compared to sodium salicylate. Simultaneously, Concurrent application of both regulators synergistically enhanced their efficacy, yielding the most favorable outcomes. In addition, this study noted that while the initial effects of these regulators were not pronounced, their sustained application significantly improved wheat growth in stressful condition and alleviated the detrimental impacts of salt stress. This approach could effectively guarantee the food security and production in China.

## Introduction

Soil salinization, a critical issue in the current agricultural production process, is an important factor affecting the global ecological environment. According to statistics, the salinized land area of the world is about 8.0 × 10^8^ hm^2^, which has suffered different degrees of salinization hazards^1,2^. Projections suggest that by 2050, over 50% of the world’s arable land will suffer from salinization, thereby constraining global agricultural output and food supply^3–5^. Soil salinization induces salt stress in plants, with sodium salt stress being particularly detrimental, comprising 45% of salinized soil’s total area. High concentration of Na^+^ disrupts the uptake and utilization of essential minerals like potassium, calcium, and zinc in plants, hence impairing efficient nutrient utilization. This also results in osmotic pressure imbalance, destruction of cell membrane structure, and leakage of intracellular substances, which directly impairs photosynthesis and reduces plant growth rate. Consequently, this affects both aboveground and underground plant structures, with a notable reduction in the fresh and dry weight of plant tissues. Specifically, the dry and fresh weight of the aboveground leaf tissues declines markedly^6–8^. Moreover, salt stress triggers ion imbalance and high osmotic stress in plant tissues, increasing ion concentration and causing ion toxicity. This leads to an imbalance in the production and removal of reactive oxygen species, resulting in their excessive accumulation and inducing variations in cellular antioxidant capacity, ultimately causing oxidative damage. In response, the intracellular antioxidant defense system is activated, producing enzymes like SOD, POD and CAT to gradually eliminate excessive ROS and mitigate external stress, playing a crucial role in plant growth and development^9–11^. Prolonged exposure to high salt levels interferes with plant metabolism and leads to an accumulation of osmotic substances. Over time, the damage from salt stress intensifies, potentially resulting in complete plant wilt or death^7,12,13^.

Wheat (*Triticum aestivum* L.), a crucial food crop globally, has its quality and yield directly impacting farmers’ income and sustainable agricultural development^14–16^. In wheat cultivation, abiotic stresses such as low temperature, heavy metals, and salinity are common, with salt stress particularly hindering high and stable yield^5,17^. Consequently, enhancing wheat’s salt tolerance remains a critical research focus in agricultural production. Research has identified two primary approaches to address soil salinization’s impact on wheat: firstly, cloning and analyzing wheat’s salt tolerance genes, and employing transgenic technology to ensure the target gene’s effective expression, resulting in salt-tolerant wheat varieties^18,19^. Meanwhile, the use of exogenous plant growth regulators directly applied to wheat to regulate physiological activity and substance content in plants, thereby boosting wheat’s salt tolerance and enhancing resistance against salt stress damage^20^.

The exogenous application of plant hormones is another effective method to cope with salt stress^21,22^. Previously studies have shown that plant growth regulators can increase plant salt tolerance, highlighting that IAA effectively regulates physiological activities in shoots and roots under salt stress, promoting their growth and development^23^. Additionally, research on melatonin (MT) reveals its role in regulating plant growth, photosynthesis, seed germination, and alleviating various stresses^24^. MT also acts as a free radical scavenger, enhances antioxidant enzyme activity^25^, and reduces plant susceptibility to cold, heat, salt, drought and other stresses^26^. Moreover, brassinolide (BR) responds to drought, salt stress, low and high temperatures, and heavy metal stress by regulating numerous enzymes and exogenous regulators, fully utilizing the plant’s growth potential and advantages in modulating growth and development^27–31^. Furthermore, studies on abscisic acid (ABA) have shown its significant role in promoting root growth and responding promptly to salt stress environments^32^. Therefore, identifying growth regulators that enhance wheat’s salt tolerance is vitally important for wheat cultivation in stressful environments, improving yields, and further securing national food security.

Folcisteine, also known as *N*-acetylthioproline (ATCA), initially served as a pharmaceutical intermediate for protecting against myocardial ischemia, and is a derivative of proline. In agriculture, it functions as a biological stimulant to enhance biological activity, cytokines, and auxin. Its applications include regulating crop cell osmotic pressure, promoting seed germination, cell division, and elongation, reducing transplant shock, protecting chlorophyll, improving nutrient utilization, maintaining water and nutrient transport balance, extending the flowering period, increasing fruit setting rate and yield, delay senescence, enhance stress resistance, and reduce fruit drop. Katrina et al. discovered that folcisteine effectively promotes flower bud differentiation and fruit development in apple trees, significantly increasing both the number and yield of fruits^33^. Furthermore, various studies have demonstrated that different concentrations of folcisteine can also enhance the growth and development of apricot trees, improve fruit retention and setting rates^34–36^, increase fruit yield and individual fruit weight^37^, and augment chlorophyll content, thereby elevating the sugar content of the fruit^38^.

Sodium salicylate, known as sodium 2-hydroxybenzoate (Na-SA), is a salicylic acid (SA) derivative with similar biological activities. Commonly, a 0.05–0.1% concentration is used for foliar spraying. Since the late 1970s, SA has been recognized for its defensive and protective functions in plants against pathogens. It can induce system acquired resistance (SAR) in plants and plays a pivotal role in SAR formation. This effect is likely due to salicylic acid acting as a key signal molecule, altering antioxidant content and enzyme activity, promoting phytoalexin, lignin, and photosynthetic pigment biosynthesis, and increasing cell wall lignification, thus enhancing stress resistance under stressful conditions^39–41^. The application of salicylic acid has been shown to improve photosynthetic pigment synthesis in cucumber seedlings under high salinity, boost photosynthesis, promote root formation, and mitigate the inhibitory effects of high salt concentrations on seedling growth^42^. Saleh et al. reported that spraying SA under high temperature stress significantly increases antioxidant enzyme activity in plants, enhancing free radical scavenging, reducing oxidative damage, and ensuring normal growth in mung bean seedlings^43^. Zanganeh et al. found that salicylic acid can regulate the absorption of beneficial elements, modulate mineral element metabolism, and promote plant growth. In maize seeds, soaking in salicylic acid effectively reduces the later-stage toxicity of Pb, improves Fe homeostasis, decreases Pb absorption, and enhances root growth and NO content under metal lead ions stress^44^. Asim et al. observed that exogenous salicylic acid mitigates the effects of prolonged drought stress in rice by reducing water loss and inducing antioxidant system production^45^.

To date, there has been no domestic research on the mitigation mechanism of the combined application of sodium salicylate and folcisteine in wheat under salt stress conditions. Thus, this study aimed to verify whether these two growth regulators could positively affect wheat growth under salt stress. An indoor hydroponic experiment was carried out, measuring the content of malondialdehyde (MDA) and active oxygen, as well as the activity of antioxidant enzymes, to preliminarily explore the mitigation mechanisms of sodium salicylate and folcisteine in wheat under salt stress.

## Materials and methods

### Cultivation and collection of experimental materials

Healthy, plump wheat seeds (Bainong 207) were selectedand soaked in a 10% H_2_O_2_ solution for 10 min, followed by three rinses with distilled water to remove residual for 12 h in the dark for germination. Post-germination, the seeds were arranged on absorbent paper soaked in saturated CaSO_4_ solution, spaced 1 cm apart, with the hairy side facing upwards, and covered with another piece of absorbent paper. The paper was then rolled up, and the seedlings were raised in a greenhouse for 7 days under light-avoiding conditions. Healthy seedlings with consistent growth were selected and transplanted into moist sponge blocks in seedling pots containing 1/2 strength Hoagland nutrient solution for hydroponic growth. Once the seedlings reached the three-leaf stage, they were transferred to full-strength Hoagland nutrient solution with 100 mmol/L NaCl added for salt stress treatment. After 12 h of treatment, folcisteine (25 mg/L) and sodium salicylate (0.05 mg/L) were applied individually by foliar spraying, diluted with distilled water to the respective concentration.

### Experimental design

The experiment was conducted in a greenhouse witha 14/10 h light/dark cycle, a light intensity of 400 μmol/m^2^/s, 60% humidity, and daytime/night temperatures of 32/26 °C. The growth and development of wheat were observed via hydroponics. Four treatment groups and one water control group were established (Table 1), each with three replicates, totaling 36 wheat seedlings. Samples were collected 3 and 6 days post-spraying, with 5 wheat plants from each treatment group selected for morphological index and biomass determination. Additionally, leaves from 6 wheat plants per treatment, forming one biological replicate for every two plants, were stored -80 °C for subsequent analysis of harmful substance content and antioxidant enzyme activity in cell tissues post-oxidative damage.

**Table 1:** Experimental treatment.

Experimental group	Experimental treatment
CK	No salt stress and spraying regulator on leaves
KB	Add salt stress with leaves sprayed with distilled water
ATCA	Add salt stress with leaves sprayed with folcisteine
Na-SA	Add salt stress with leaves sprayed with sodium salicylate
Na-SA + ATCA	Add salt stress with leaves sprayed with folcisteine and sodium salicylate in the meantime

Each experimental treatment was repeated three times in parallel.

### Determination of morphological indexes and biomass accumulation of wheat

For the analysis of wheat morphology and biomass, each plant was carefully divided into aerial and root parts using a dissecting knife at the rhizome junction. The separated leaves and roots were spread out on the scanning table of the Epson Expression 12,000 XL scanner. Morphological indexes, including leaf projection area, total root length, total root area and average root diameter, were analyzed usingWinRHIZO Pro 2017 root image analysis software. The average plant height was calculated from three parallel measurements of the aboveground parts post-shearing.

The separated sections of the wheat plant were blotted with absorbent paper to remove surface moisture and then weighed for their fresh weight. Subsequently, they were placed in kraft paper bags and subjected to deactivation in a drying oven at 105 °C for 30 min, followed by drying at 80 °C for 6 h until a constant weight was achieved, and then the dry weight was recorded^46^.

### Determination of reactive oxygen species (ROS) content

#### Determination of hydrogen peroxide (H_2_O_2_) content

The H_2_O_2_ level was gauged by assessing the absorbance of the purple complex formed by Fe^3+^ and xylenol orange^47^. This involved weighing 0.1 g of cut leaves and adding 600 μL of pre-cooled (-20 °C) acetone to form a homogenate. The mixture underwent high-speed refrigerated centrifugation at 4 °C for 15 min at 10,000×*g*, after which the supernatant was collected for analysis.

The reaction mixture consisted of: 200 μL of distilled water, 500 μL of 400 mM sorbitol, 500 μL of 0.8 mM ammonium ferrous sulfate, and 500 μL of 0.4 mM xylenol orange dissolved in 25 mM sulfuric acid, mixed in a 2 mL centrifuge tube. Adding 300 μL of the supernatant to this mixture, it was then incubated at 30 °C for 30 min, and the OD560 value was measured.

A standard curve was prepared using varying concentrations of H_2_O_2_ (0, 60, 120, 180, 240, 300 μL of 100 μM H_2_O_2_), along with 750 μL each of 400 mM sorbitol, 0.8 mM ammonium ferrous sulfate, and 0.4 mM xylenol orange, incubated at 30 °C for 30 min. The OD560 value of this standard curve was used to calculate the actual H_2_O_2_ content in the sample.

#### Determination of superoxide anion (O^2−^) content

O^2−^ levels were measured using the method proposed by Erstner et al.^48^. A 0.1 g sample of fresh weight was combined with 1 mL of 50 mM PBS (pH 7.8) and ground. The mixture was centrifuged at 4 °C for 20 min at 10,000×*g*, and the supernatant was collected for analysis.

The reaction system included 50 μL of 6.25 mM PBS (pH 7.8), 100 μL of 0.25 mM hydroxylamine hydrochloride and 50 μL of supernatant. This mixture was incubated in a 25 °C water bath for 1 h to generate nitrite. A control was prepared using an equal volume of PBS in place of the supernatant. Subsequently, 100 μL of 4.25 mM sulfanilic acid and 100 μL of 1.75 mM α-naphthylamine were added, followed by incubation in the dark at 25 °C for 20 min. The absorbance of the reaction mixture at 530 nm was then measured.

Sodium nitrite (NaNO_2_) solutions were prepared at concentrations of 0, 10, 20, 30, 40 and 50 μM to construct a standard curve equation. The actual content of superoxide anion (O^2−^) in the samples was calculated using this the standard curve.

#### Determination of antioxidant enzyme activity

The crude enzyme extract was prepared following the method described by Zhang et al.^49^. Initially, the sample was treated with liquid nitrogen and ground using a freezing grinder. Subsequently, 0.1 M PBS (pH 7.5) containing 1 mM EDTA and 1% PVPP was added. The mixture was then oscillated, homogenized, and centrifuged at 15,000×*g* at 4 °C for 20 min. The supernatant obtained was reserved for later use.

#### Determination of superoxide dismutase (SOD) activity

SOD activity (EC 1.15.1.1) was measured using the NBT photoreduction method^50^. The reaction mixture, composed of 20 μL of 130 mM methionine solution, 20 μL of 30 μM EDTA-Na_2_ solution, 135 μL of 50 mM PBS(pH 7.8), and 20 μL of 0.75 mM NBT solution, was prepared in a 2 mL centrifuge tube. Then 5 μL of the crude enzyme extract was added. After shaking, the mixture was placed under 4000 lx light at 25 °C for 20 min. The control group replaced the enzyme extract with 50 mM PBS. The absorbance at 560 nm was subsequently measured in the dark to assess SOD activity.

#### Determination of peroxidase (POD) activity

POD (EC1.11.1.7) activity was determined using the guaiacol method^51^. 28 μL of guaiacol was added to 50 mL of 100 mM phosphate buffer (pH 6.5) and dissolved by heating. Once cooled, 19 μL of 30% H_2_O_2_ solution was mixed into the solution. A total of 100 μL of this reaction mixture and 100 μL of crude enzyme extract were added to a centrifuge tube. The absorbance change at 470 nm was measured every 30 s for 3 min, with the change within the first minute representing the enzyme activity.

#### Determination of catalase (CAT) activity

CAT (EC1.11.1.6) was determined following the method outlined by Chen et al.^52^. A 20 μL enzyme extract sample was added to 4.98 mL of 100 mM phosphate buffer (pH 7.0), followed by the addition of 50 mM 30% H_2_O_2_. The absorbance change of H_2_O_2_ at 240 nm was measured over 1 min to determine the enzyme activity.

### Statistical analysis

Data collected were initially analyzed using ANOVA with SPSS software (Ver. 17.0, SPSS Inc.). Statistical differences were determined using Fisher’s least significant difference test (*α* = 0.05). Graphpad Prism (Ver. 9.0.0) was utilized for data graphing.

### Ethics declarations

The collection of plant materials is in accordance with relevant institutions, national and international norms and legislation in the study.

## Results

### Morphological growth index and biomass accumulation

Following the application of plant growth regulators to wheat leaves, the observed plant height and total root length under salt stress exceeded those of the control group (CK) and water comtrol group (KB), suggesting that sodium salicylate and folcisteine significantly alleviate growth inhibition in wheat. This trend was consistent at both 3 and 6 days post-treatment (Table 2). After 3 days, the plant height in the ATCA group, sodium salicylate (Na-SA) group, and combined Na-SA and ATCA group increased by 10%, 24%, and 36% respectively compared to the KB group. Total root length in these groups increased by 16%, 42%, and 44%, respectively. The leaf projection area was higher in the Na-SA and Na-SA + ATCA groups than in the KB group, with significant differences observed. The ATCA group showed slight improvement in leaf projection area, albeit marginally lower than the KB group. Leaf projection area in the Na-SA and Na-SA + ATCA groups increased by 11% and 22%, respectively, relative to the KB group. At 6 days, each treatment group exhibited an increase in leaf projection area compared to the KB group. Specifically, the ATCA, Na-SA, and Na-SA + ATCA groups showed increases of 8%, 21%, and 36%, respectively, with notable differences.

**Table 2:** Effects of sodium salicylate and folcisteine on the growth and development of wheat seedlings.

Time of treatment (days)	Treatment	Plant height (cm)	Total root length (cm)	Leaf projection area (cm^2^)	Root average diameter (cm)	Root surface (cm^2^)
3	CK	19.37 ± 1.06bc	943.5 ± 6.9ab	14.42 ± 0.88ac	0.340 ± 0.022a	104.4 ± 2.4ab
	KB	16.67 ± 0.69c	839.3 ± 69.7b	12.39 ± 0.90bc	0.304 ± 0.018ab	86.2 ± 11.1b
	ATCA	18.40 ± 0.55bc	969.4 ± 106.4ab	10.42 ± 0.55c	0.323 ± 0.008b	98.3 ± 9.99ab
	Na-SA	20.63 ± 0.61ab	1195.5 ± 128.7a	13.77 ± 0.96ab	0.324 ± 0.002b	121.4 ± 12.2a
	Na-SA + ATCA	22.33 ± 1.07a	1205.4 ± 17.4a	15.08 ± 0.06a	0.392 ± 0.006ab	123.3 ± 2.3a
6	KB	20.96 ± 0.64bc	1660.1 ± 153.7bc	23.34 ± 0.95ab	0.325 ± 0.020bc	102.6 ± 4.1a
	CK	19.23 ± 0.26c	1498.6 ± 87.8c	18.82 ± 1.03b	0.309 ± 0.008c	93.3 ± 2.1a
	ATCA	21.90 ± 0.78b	1662.1 ± 50.8bc	20.34 ± 0.51b	0.338 ± 0.003ab	99.6 ± 3.2c
	Na-SA	23.07 ± 1.18b	2011.3 ± 204.7ab	22.87 ± 2.06ab	0.337 ± 0.005ab	129.2 ± 0.2b
	Na-SA + ATCA	26.53 ± 0.78a	2236.9 ± 140.4a	15.08 ± 1.73a	0.428 ± 0.009a	131.7 ± 7.9b

The lowercase letters in the same column in the table indicate their differences under the significance level of P < 0.05.

The root surface area in all three experimental treatment groups significantly exceeded that of the KB group (Table 2). At 3 days, root surface areas in the ATCA, Na-SA and Na-SA + ATCA groups increased by 14%, 41% and 43% respectively, compared to the KB group. However, the root surface area in the ATCA group was lower than in the CK group, with the Na-SA + ATCA group demonstrating the most significant effect. At 6 days post-application, the root surface areas in the ATCA, Na-SA, and Na-SA + ATCA groups had increased by 0.3%, 39%, and 33%, respectively, in comparison with the KB group. The root surface area of the ATCA group remained lower than that of the CK group, indicating a non-significant mitigation effect on wheat growth. Overall, the Na-SA group exhibited the best performance.

At 3 days post-application, the effect of the growth regulators in the ATCA group and the Na-SA group on wheat under salt stress was not pronounced compared to KB group. The increase in average root diameter was marginal, with the ATCA and Na-SA groups showing increases of 6.25% and 6.27%, respectively, compared to the KB group, but still lower than the control group (CK) (Table 2). The combined Na-SA + ATCA group demonstrated the most significant improvement, with increases of 30% and 15% compared to the KB and CK groups, respectively. By 6 days, significant differences emerged among the treatments, with the Na-SA + ATCA group performing the best, followed by the Na-SA group. Overall, the regulators, when used individually, had a limited effect on the lateral growth of wheat roots, while their combined application notably enhanced root growth, significantly outperforming the single regulator treatment.

Three days after spraying regulators, the physiological characteristics of wheat under salt stress showed significant improvement compared to the CK and KB groups (Table 3). Notably, the fresh weight of aboveground parts and dry weight of underground parts differed significantly among treatments. The ATCA, Na-SA, and Na-SA + ATCA groups showed increases in aboveground fresh weight by 5%, 26% and 32%, respectively. There were no noticeable differences in the fresh weight of underground parts and the dry weight of aboveground parts. The Na-SA and Na-SA + ATCA groups had a substantial mitigating effect on wheat growth under stress, whereas the ATCA group alone did not significantly improve the wheat’s stress environment. The Na-SA + ATCA group exhibited the most effective growth mitigation across all growth indices of wheat, surpassing the effects of single regulator treatments, suggesting a synergistic effect between the two regulators. Overall, it can be inferred that both growth regulators influence the growth and development of wheat under salt stress.

**Table 3:** Effects of sodium salicylate and folcisteine on biomass accumulation of wheat.

Time of treatment (days)	Treatment	Aboveground fresh weight (g)	Underground fresh weight (g)	Aboveground dry weight (g)	Underground dry weight (g)
3	CK	0.701 ± 0.05ab	0.819 ± 0.06a	0.147 ± 0.005a	0.064 ± 0.007ab
	KB	0.597 ± 0.07b	0.743 ± 0.05a	0.124 ± 0.011a	0.058 ± 0.005b
	ATCA	0.628 ± 0.05ab	0.751 ± 0.06a	0.152 ± 0.027a	0.073 ± 0.007ab
	Na-SA	0.750 ± 0.04ab	0.947 ± 0.14a	0.140 ± 0.014a	0.083 ± 0.009ab
	Na-SA + ATCA	0.790 ± 0.02a	1.038 ± 0.10a	0.171 ± 0.020a	0.085 ± 0.003a
6	KB	1.091 ± 0.114b	0.91 ± 0.094ab	0.135 ± 0.014ab	0.094 ± 0.003ab
	CK	0.927 ± 0.020b	0.77 ± 0.054a	0.115 ± 0.007c	0.053 ± 0.007c
	ATCA	1.202 ± 0.074b	0.99 ± 0.030a	0.142 ± 0.008bc	0.083 ± 0.009bc
	Na-SA	1.165 ± 0.096b	1.04 ± 0.047a	0.160 ± 0.026ab	0.100 ± 0.018ab
	Na-SA + ATCA	1.867 ± 0.172a	1.09 ± 0.079a	0.189 ± 0.03a	0.117 ± 0.012a

The lowercase letters in the same column in the table indicate their differences under the significance level of P < 0.05.

With the prolonged action of the regulators, wheat’s physiological characteristics generally improved significantly, with each treatment showing marked differences (Table 3). Compared to 3 days, the aboveground fresh weight and underground dry weight in the ATCA group increased by 91% and 14%, respectively, after 6 days. The Na-SA group saw increases of 55% and 20%, respectively, while the Na-SA + ATCA group rose by 136% and 38%, respectively. Notably, significant differences were observed in the underground fresh weight and aboveground dry weight of wheat. In the ATCA group, the underground fresh weight increased by 32%. In the Na-SA group, the increases were 10% and 13%, respectively, and in the Na-SA + ATCA group, they were 5% and 11%, respectively. It was concluded that the mitigating effect of Na-SA on wheat under salt stress was markedly superior to that of ATCA, and the combined treatment of both regulators significantly outperformed single regulator treatments, demonstrating a notable synergistic effect.

### The concentration of malondialdehyde (MDA)

**Figure 1:**
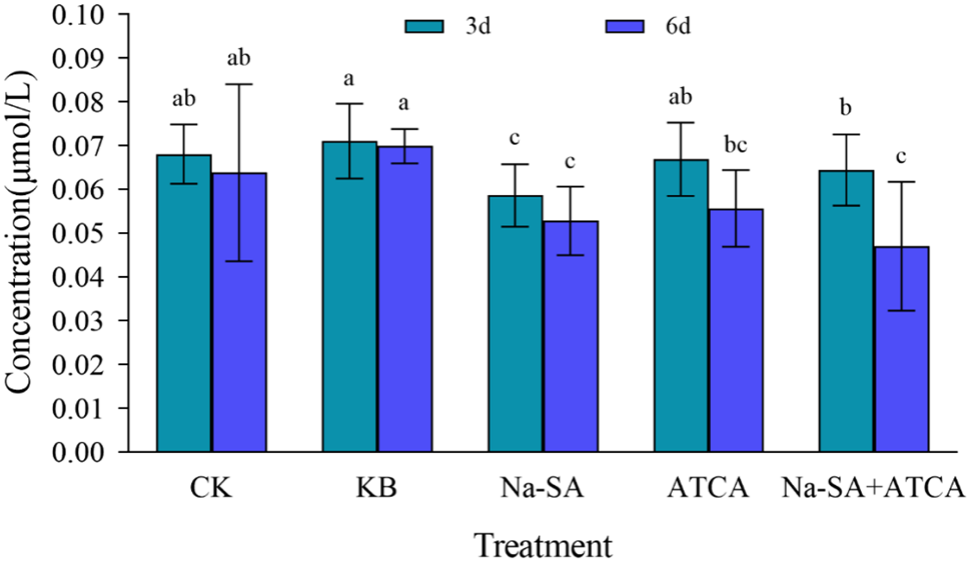
MDA concentration in wheat leaves.

The study revealed that after 3 days of treatment, the concentration of malondialdehyde (MDA) in the wheat leaves of ATCA group, Na-SA group and combined Na-SA + ATCA group was significantly lower than that in both the KB and CK groups. Specifically, the ATCA group showed a reduction of 3% and 7% compared to the KB and CK groups, respectively. The Na-SA group experienced a decrease of 15% and 18%, respectively, while the Na-SA + ATCA group showed a 6% and 10% reduction. The Na-SA group exhibited the most pronounced regulatory effect, suggesting that the regulator began mitigating the salt stress in wheat and reducing leaf tissue cell damage, thereby decreasing MDA concentrations in the plant. After 6 days, the impact of single regulator treatments on wheat did not significantly change. The ATCA group’s MDA concentration reduced by 13% and 20% compared to the KB and CK groups, respectively. The Na-SA group showed a 17% and 25% reduction, and the Na-SA + ATCA group experienced a decrease of 25% and 32%, respectively, substantially lower than the single regulator treatment groups. The Na-SA + ATCA group had the most significant effect. Overall, MDA concentrations in the ATCA, Na-SA, and Na-SA + ATCA groups decreased by 17%, 10%, and 27%, respectively, after 6 days compared to 3 days (Fig. 1). Thus, the combined Na-SA + ATCA group had the most effective outcome, with significant differences observed among the experimental groups.

### The content of reactive oxygen species (ROS)

#### The content of hydrogen peroxide (H_2_O_2_)

**Figure 2:**
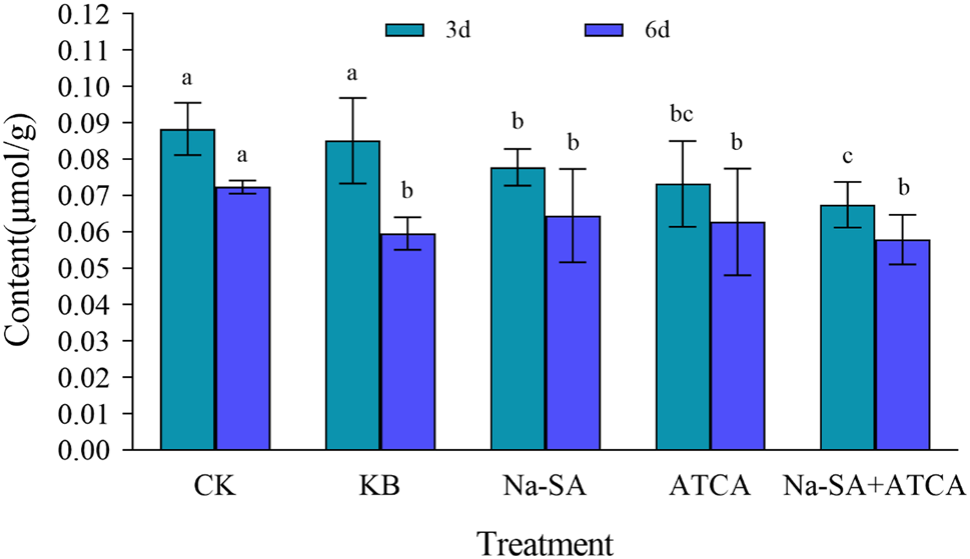
H_2_O_2_ content in wheat leaves.

The results indicated that wheat leaf tissue produced a large amount of H_2_O_2_ under salt stress. Three days post-regulator foliar spraying, the H_2_O_2_ content in the ATCA, Na-SA and Na-SA + ATCA groups was considerably lower than in the CK and KB groups, with the Na-SA + ATCA group exhibiting the best result. This group showed a 25% and 22% reduction in H_2_O_2_ content compared to the CK and KB groups, respectively. After 6 days of foliar spraying, H_2_O_2_ content gradually decreased, with reductions of 14%, 11%, and 15% in the ATCA, Na-SA, and Na-SA + ATCA groups, respectively (Fig. 2). The continuous application of the two regulators showed that the ATCA group’s performance surpassed that of the Na-SA group, and the Na-SA + ATCA group was the most effective, indicating a clear synergistic effect of the two regulators in promoting wheat growth under salt stress.

#### The content of superoxide anion (O^2−^)

**Figure 3:**
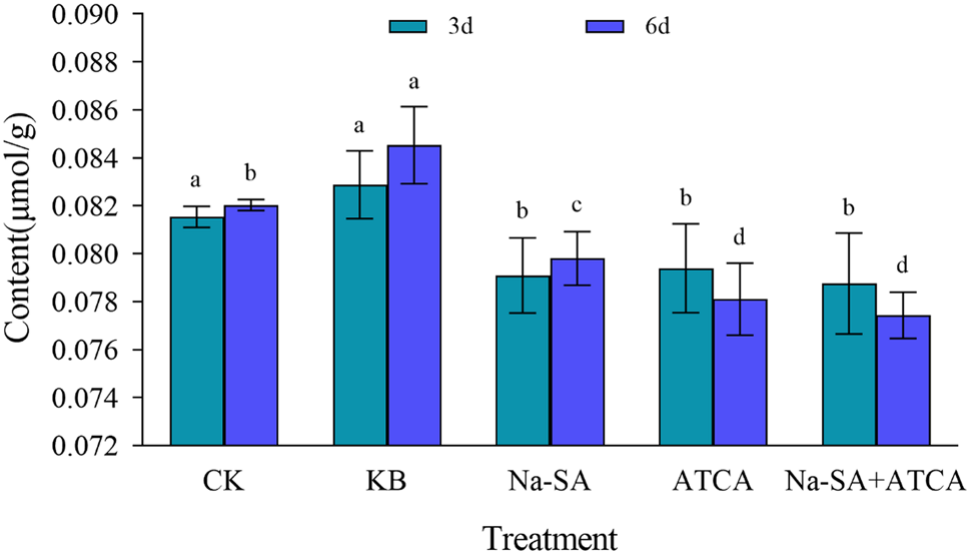
O^2−^ content in wheat leaves.

The results showed that the content of superoxide anion (O^2−^) in wheat leaves of KB group was significantly higher than that of the other four treatment groups, indicating that high concentration of salt caused great damage to wheat leaf tissue cells, resulting in a large accumulation of superoxide anion in cell fluid, inhibiting the normal growth and development of wheat (Fig. 3). Following treatment with two regulators, the O^2−^ content in the wheat leaves was markedly lower than in both the CK and KB groups, with a noticeable difference between the experimental groups. At 3 days, the O^2−^ content in both the ATCA group and Na-SA group was approximately the same, showing a 1% and 4% reduction compared to the CK and KB groups, respectively. Na-SA + ATCA group exhibited the lowest O^2−^ content, with reductions of 5% and 3% compared to the CK and KB groups, respectively. At 6 days, the initial regulatory effect of the ATCA and Na-SA groups was less apparent, and no differences were observed between the treatments. Later on, the ATCA group increasingly mitigated O^2-^accumulation. The regulatory effect of the ATCA group was 1.2 times that of the Na-SA group. The Na-SA + ATCA group maintained the best performance, significantly repairing wheat tissue cell damage and reducing intracellular superoxide anion accumulation.

### The activity of Antioxidant enzyme

#### The activity of superoxide dismutase (SOD)

**Figure 4:**
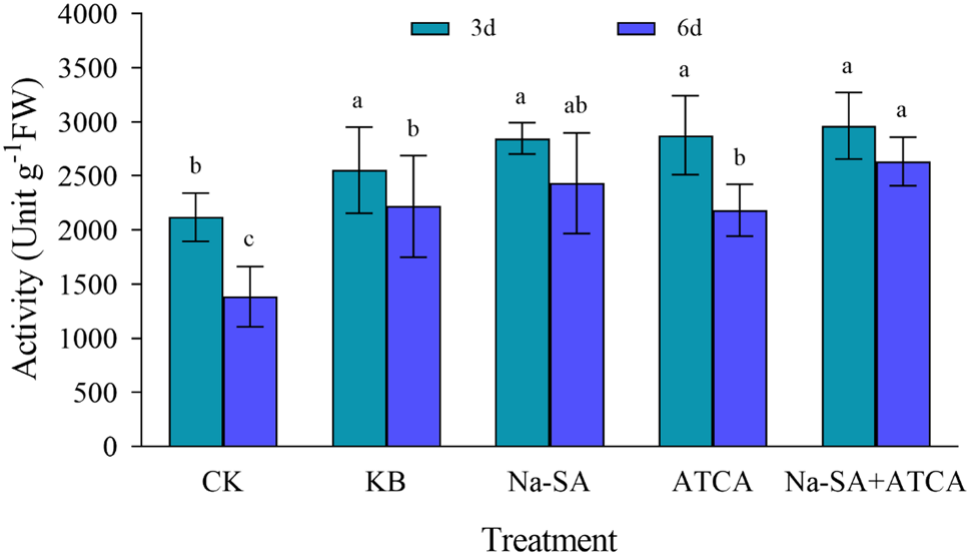
SOD activity in wheat leaves.

The results indicated that at 3 days, the SOD activity in the CK group was the lowest, while the enzyme activity in other treatment groups exceeded the CK group by more than 1.7 times, with no significant differences among these groups. The ATCA, Na-SA, and Na-SA + ATCA groups exhibited increases in enzyme activity by 75%, 76%, and 80% respectively compared to the CK group, demonstrating the regulators’ significant mitigation effect on wheat growth under salt stress. By 6 days, the differencs in enzyme activity among the treatments were substantial. The CK group still had the lowest SOD activity. The Na-SA group’s enzyme activity was 1.1 times that of the ATCA group, and the Na-SA + ATCA group had the highest enzyme activity, 1.2 times that of the Na-SA group. The ATCA, Na-SA, and Na-SA + ATCA groups showed increases of 121%, 138%, and 158% in enzyme activity, respectively, compared to the CK group (Fig. 4). Overall, the application of the two regulators indicated that the combined treatment was more effective than single treatments, followed by the Na-SA group.

#### The activity of peroxidase (POD)

**Figure 5:**
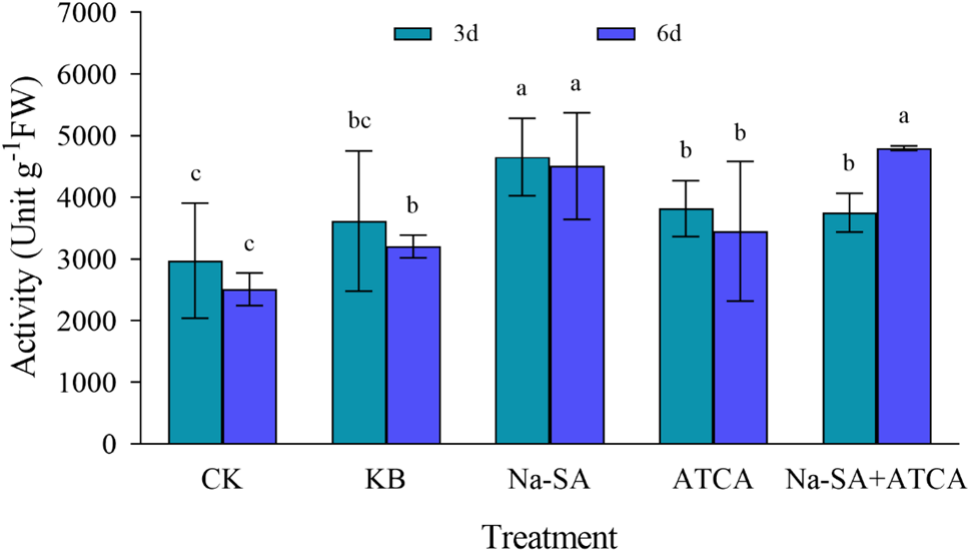
POD activity in wheat leaves.

At 3 days post-treatment, the peroxidase activity in the Na-SA group was the highest and significantly differed from the other treatment groups. The activity in the Na-SA group was 1.1 times and 1.3 times higher than in the Na-SA + ATCA and ATCA groups, respectively. Compared to the CK and KB groups, the activity in the Na-SA group increased by 36% and 12%. Additionally, the enzyme activity in the other experimental treatment groups was higher than in the CK group. By 6 days, the wheat in the Na-SA + ATCA group exhibited the highest peroxidase activity, with the Na-SA + ATCA group showing an activity 1.2 times that of the Na-SA group, and 101% and 46% higher than the CK and KB groups, respectively (Fig. 5). Over time, the Na-SA + ATCA group maintained the highest enzyme activity, followed by the Na-SA group, which continued to exert a significant regulatory role.

#### The activity of catalase (CAT)

**Figure 6:**
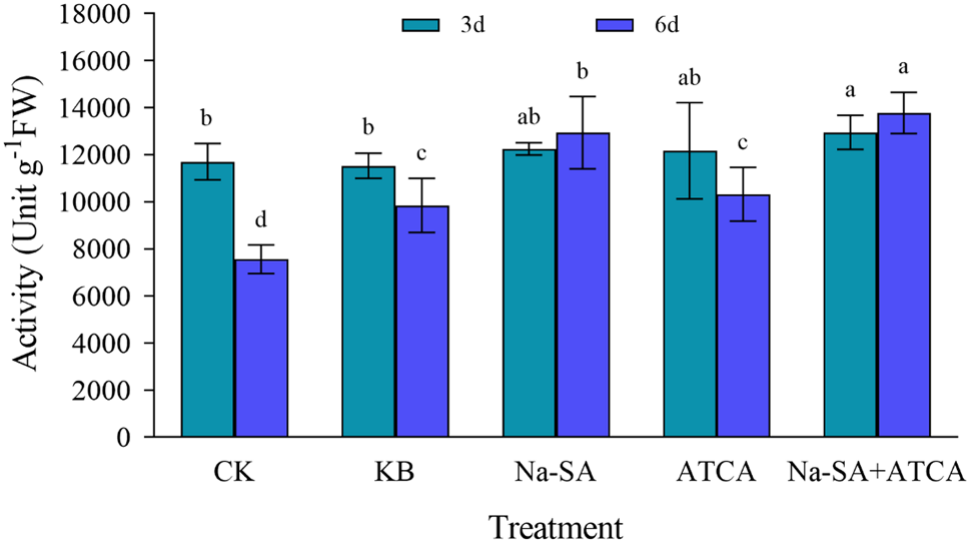
CAT activity in wheat leaves.

At 3 days, the overall trend indicated minimal differences in catalase activity among the treatment groups. The ATCA, Na-SA, and Na-SA + ATCA groups displayed increases in enzyme activity by 4%, 5%, and 11% respectively, compared to the CK group, suggesting that the regulators had a mitigating effect on wheat growth. At 6 days, the enzyme activities in the ATCA, Na-SA, and Na-SA + ATCA groups differed significantly from those in the CK and KB groups (Fig. 6). The activity in these three treatment groups was 1.6 times higher than in the CK group and 1.3 times higher than in the KB group. Furthermore, significant differences were observed between the treatments. The enzyme activities in the ATCA, Na-SA, and Na-SA + ATCA groups increased by 59%, 71% and 82%, respectively compared to the CK group. Overall, it can be deduced that with the continuous influence of the regulators, their regulatory effect becomes increasingly pronounced. The Na-SA + ATCA group emerged as the most effective in regulating wheat growth, followed by the Na-SA group.

## Discussion

When crops are exposed to salt stress, a high concentration of salt initially exerts a significant inhibitory effect on seedling growth and development, directly impacting the accumulation of dry matter and leading to a marked reduction in leaf growth rate^6^. Studies have shown that increasing salt concentration hinders the biomass of roots, shoots, and leaves of crops, thereby significantly diminishing biomass accumulation^7^. Under the environmental pressure of high concentrations, plants accumulate large quantities of ROS, which damage cell membranes, significantly diminish cell antioxidant capacity, and cause oxidative cell damage. This accumulation also impairs the activity of antioxidant enzymes, affecting plant metabolism and severely restricting normal plant growth and development^9,10^.

During normal growth, plants subjected to environmental stress experience inhibited root and leaf growth^53^. Changes in crop morphological characteristics can directly indicate the extent of salt damage^54^. A suitable leaf area index and biomass at the seedling stage are advantageous for grain filling and yield enhancement in later stages. In this study, the combined treatment of ATCA and Na-SA significantly outperformed single regulator treatments in terms of morphological and physiological indexes, and biomass accumulation in wheat under salt stress. The effect of Na-SA alone surpassed that of ATCA, aligning with findings by Mimouni et al.^55^, which indicated that tomato plants exposed to salt stress and treated with 0.01 mM SA exhibited enhanced growth parameters such as branch and root dry weight, and leaf area. Notably, the alleviating effect of regulators on the longitudinal growth of wheat roots was more pronounced than on their transverse growth, with the root morphology generally appearing slenderer under the regulation of the root system.

Salt stress disrupts the dynamic equilibrium of active oxygen scavenging mechanisms in plant cells, leading to the accumulation of ROS. Prolonged inability to effectively eliminate these ROS can cause oxidative damage to plant cells, ultimately resulting in the degradation of cellular membrane systems, proteins, and photosynthetic systems^56^. To counteract external stress, plants activate their defense mechanisms, producing a plethora of antioxidant enzymes and antioxidants that engage in redox reactions with ROS. These include SOD, CAT, POD, which are pivotal in effectively removing ROS and minimizing plant damage^57^. In this study, foliar spraying with ATCA and Na-SA significantly reduced the accumulation of O^2−^ and H_2_O_2_ in wheat leaves, repairing oxidative damage to wheat tissue cells. The regulatory effect of ATCA was marginally superior to that of Na-SA. Moreover, the antioxidant enzyme activity in the plants indicated that the regulatory adjustment under stress conditions was initially gradual, with minimal variation in enzyme activity across treatments during the early stages post-spraying. This suggests that the initial application may have reached a threshold of action, rendering the impact of the plant growth regulator less pronounced overall. However, subsequent applications led to significant differences in enzyme activity among treatments. The combined treatment with ATCA and Na-SA exhibited a markedly better regulatory effect on each enzyme activity than single regulator treatments. The efficacy of Na-SA alone surpassed that of ATCA, aligning with findings by Chen et al.^58^ and Sihag et al.^59^ on *Sorghum bicolor* L. seedlings under chromium stress and Leymus chinensis seeds under salt stress, respectively, when treated with salicylic acid through foliar spraying (0.5 mM) and seed soaking (0.1 mM). This demonstrates that the two regulators significantly enhance antioxidant enzyme activity in wheat under salt stress, effectively mitigating the stress and ensuring normal growth and development.

Salt stress is known to cause damage to the plasma membrane, increase its permeability, lead to intracellular electrolyte leakage, and elevate electrical conductivity^60^. The breakdown of the plasma membrane results in the accumulation of malondialdehyde^61,62^. MDA is a critical important indicator of plant cell response to stress and cell membrane damage. In this study, the concentration of MDA was significantly reduced following Na-SA leaf spray treatment, demonstrating the most effective regulation and substantially alleviating the salt stress in wheat. This reduction in MDA concentration reduced leaf tissue cell damage. These findings align with those of Chen et al.^58^ and Manaa et al.^63^, who observed that salicylic acid under salt stress significantly lowered MDA concentrations in Leymus chinensis seeds and tomato plants. At later stages of treatment, the impact of the regulators was remarkably significant, with the Na-SA treatment exhibiting the strongest effect. This is consistent with the research of Pan et al.^64^ on cucumbers under low-temperature stress, which showed that 1.0 mM SA significantly reduced MDA content, increased antioxidant enzyme activity, and induced cold tolerance in cucumber. Interestingly, our study found that the combined treatment of ATCA and Na-SA significantly outperformed single regulator treatments in regulating MDA concentration, suggesting a synergistic effect^64^.

## Conclusions

In conclusion, external salt stress considerably hampers the normal growth and development of wheat. The two plant growth regulators, ATCA and Na-SA, demonstrated varying degrees of alleviation on wheat growth under salt stress. Among the indicators examined, including morphological attributes, biomass accumulation, antioxidant enzyme activity, MDA concentration, and ROS content, the combined ATCA and Na-SA group was markedly superior to single regulator treatments, indicating a synergistic effect. This suggests that the combined application of these two growth regulators can significantly mitigate the effects of salt stress on wheat growth and development. The likely mechanism is that the regulators first decrease MDA content in plants by boosting antioxidant enzyme activity, thereby significantly reducing ROS accumulation in plant tissue and ultimately alleviating oxidative damage to cell membranes. This protection of chlorophyll content in plant leaves ensures sufficient photosynthesis for organic matter synthesis, crucial for plant growth and development. Thus, these growth regulators can effectively regulate wheat’s growth under stress conditions and alleviate growth inhibition caused by salt stress.

## Data Availability

The datasets used and/or analysed during the current study are available from the corresponding author on reasonable request.
